# Impact of the association between education and obesity on diabetes-free life expectancy

**DOI:** 10.1093/eurpub/ckad153

**Published:** 2023-08-23

**Authors:** Henrik Brønnum-Hansen, Michael Davidsen, Ingelise Andersen

**Affiliations:** Department of Public Health, Faculty of Health and Medical Sciences, University of Copenhagen, Copenhagen, Denmark; National Institute of Public Health, University of Southern Denmark, Copenhagen, Denmark; Department of Public Health, Faculty of Health and Medical Sciences, University of Copenhagen, Copenhagen, Denmark

## Abstract

**Background:**

The purpose of the study was to quantify the association between body weight and health by estimating the expected lifetime with and without diabetes (diabetes-free life expectancy) at age 30 and 65. In addition, the diabetes-free life expectancy was stratified by educational level.

**Methods:**

Life tables by sex, level of education and obese/not obese were constructed using nationwide register data and self-reported data on body weight and height and diabetes from the Danish National Health Survey in 2021. Diabetes-free life expectancies were estimated by Sullivan’s method.

**Results:**

The difference in life expectancy between not obese 30-year-old men with a long and a short education was 5.7 years. For not obese women, the difference was 4.1 years. For obese men and women, the difference in life expectancy at age 30 was 7.0 and 5.2 years. Women could expect more years without and fewer years with diabetes than men regardless of body weight and educational level. Diabetes-free life expectancy differed by 6.9 years between not obese 30-year-old men with a short and a long education and by 7.7 years for obese men with a short and a long education. For women, the differences were 5.9 and 6.6 years.

**Conclusion:**

The results demonstrate an association of obesity and educational level with life expectancy and diabetes-free life expectancy. There is a need for preventive efforts to reduce educational inequality in life expectancy and diabetes-free life expectancy. Structural intervention will particularly benefit overweight people with short education.

## Introduction

Diabetes is a socially unequally distributed disease with increasing prevalence.^1^ Diabetics have a higher mortality rate than the average population^2^ and diabetes contributes to the social inequality in mortality.^3^ Obesity is strongly associated with the risk of diabetes.^4^

Expected years of life without diabetes has been estimated in a few studies.^5–10^ Among these studies, the role of obesity in the USA was estimated by Cunningham *et al*.^7^ who found that although life expectancy increased from 1980 to 2000, diabetes-free life expectancy decreased. Obese individuals experienced the greatest loss of lifetime without diabetes. Bender *et al*.^8^ showed that diabetes was a main source of health inequality related to educational differences in overweight and obesity in Denmark.

Mathisen *et al*.^11^ have shown that differential exposure to overweight/obesity contributes to educational inequality in the incidence of type 2 diabetes. But as far as we know, the social inequality in diabetes-free life expectancy among obese and not obese people has not been investigated.

While life expectancy is a simple aggregate age-standardized indicator of mortality, health expectancy indicators add information on health that quantifies the quality of life expectancy (e.g. disease-free life expectancy and disability-free life expectancy). Health expectancy can be further stratified into different risk factor exposure levels and/or socioeconomic positions. Health expectancy by level of education is usually measured among people aged 30 years (age after completing education) and 65 years to illustrate the potential difference between young adults and older adults.

The purpose of the study was to quantify the impact of obesity on health by estimating expected lifetime without and with diabetes at ages 30 and 65 and to estimate educational inequality in life expectancy and diabetes-free life expectancy. The study thus estimated how many years one could expect to live without and with diabetes in groups characterized by body weight and educational level.

The results may improve our understanding of how a highly prevalent health determinant and health condition contribute to the social inequality in life expectancy and a disease-specific measure of health expectancy.

## Methods

**Figure 1:**
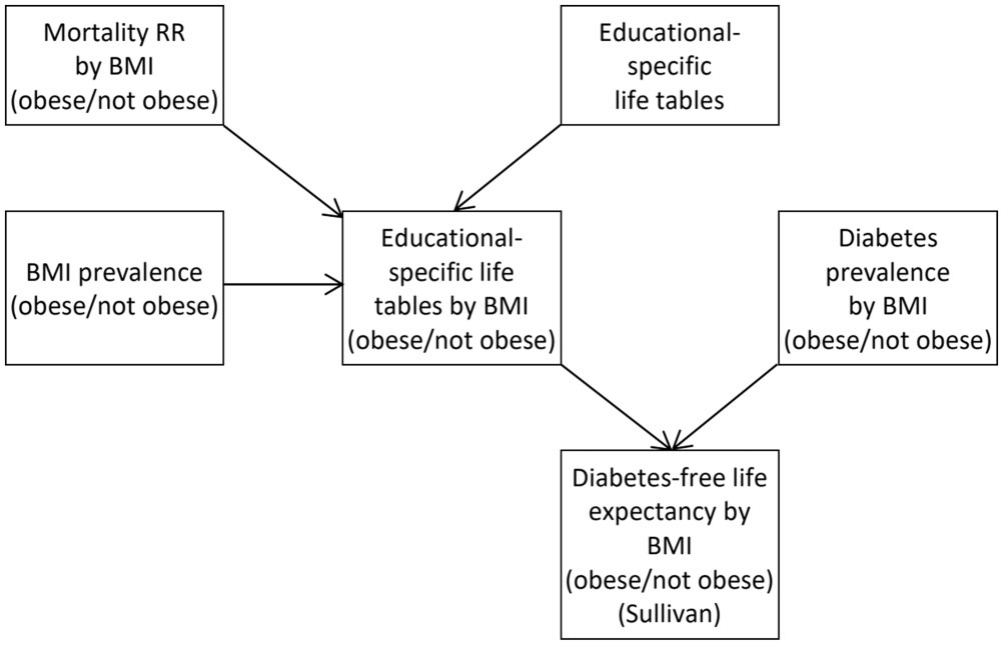
Model diagram

The study was based on nationwide register data on all Danes and data from the Danish National Health Survey (DNHS) collected in 2021 on Danes aged 16 years or older. Details and steps of the calculations are described below and summarized in the model diagram shown in figure 1.

### Construction of educational-specific life tables

Information on the highest level of completed education for all Danish citizens is reported annually and systematically to the Ministry of Education.^12^ These data and mortality data are stored at Statistics Denmark. Assuming that most people have finished their education at the age of 30, and using the unique identification code assigned to all Danes, the highest attained education and date of death were linked at the individual level for all aged 30+, and age- and sex-specific death rates by educational level were calculated for the period 2020–21. Life tables were then created for each of three levels of education according to the international standard classification of education (ISCED): Short—Primary and lower secondary education (ISCED 0-2); Medium—Upper secondary education (ISCED 3-4); Long—Tertiary education (ISCED 5-8) (http://uis.unesco.org/en/topic/international-standard-classification-education-isced).

### Survey data on educational-specific prevalence of obesity and diabetes

In 2021, a representative sample of 324 000 people was invited to DNHS, of which 56.7% participated.^13^ Self-reported prevalence of body weight and height as well as diabetes by age, sex and education were extracted from DNHS. These data were weighted according to various register information to take into account different sampling probabilities and differential non-response.^13^

Body mass index (BMI) was calculated based on questions about body weight and height. Obesity was defined by BMI of more than 30 kg/m^2^.

Information on diabetes was based on the question ‘For each of the following diseases and health problems, please indicate whether you have it or have had it in the past. If you have had it in the past, please also state whether you have any after effects’. One of the specific diseases is diabetes. However, no distinction is made between type 1 and type 2 diabetes in the question-wording. The prevalence of diabetes in Denmark is about 350 000 of which <10% suffers from type 1 diabetes.^14^

Information about education was assessed through questions about school, youth education and higher education attainment and grouped into the three levels described above.

### Construction of educational-specific life tables by obesity

Sex-, educational level- and BMI level-specific life tables were constructed using sex-, educational level- and age-specific (5-year age intervals) population death rates and prevalence of obesity. Thus, for each educational level, the sex- and age-specific death rate for not obese, d_0_, was found by solving the equation *d* = (P×RR + (1−P)) × *d*_0_. Here, the sex- and age-specific death rate, d, was readily accessible from the educational-specific life table, P was the sex- and age-specific prevalence of obesity and RR was the relative risk of death for obese individuals which were estimated from register data linked with data from DNHS and the Danish Health and Morbidity Survey (DHMS)^15^ assuming no differences between educational levels (see Table 9.4.1 in Ref. 15). The sex- and age-specific death rate for obese individuals is RR × d_0_ and the life tables for obese and for not obese individuals were constructed. (Basic data for life table construction by education and BMI are shown in the Supplementary material.)

### Estimation of educational-specific diabetes-free life expectancy by obesity

Diabetes-free life expectancy was calculated by the prevalence-based life table method suggested by Sullivan.^16^ Thus, for each combination of educational level, BMI group (obese and not obese) and sex, the expected number of years lived in 5-year age groups from 30–34 to 80–84 and 85+ years were multiplied by age-specific proportions of people without/with diabetes and expected lifetime without/with diabetes at age 30 was then calculated by adding up these years and dividing the sum by the number of survivors at age 30 (similar for age 65). By relating years without diabetes to life expectancy, the proportion of diabetes-free life expectancy was established. Death rates by educational level were not affected by sample errors as they were provided from nationwide registers for the entire population. Thus, the only source of random variation was assumed to arise from DNHS. Confidence intervals (95% CIs) were estimated from the formulae suggested by the International Network on Health Expectancy.^17^ The method for calculating health expectancy by risk factor exposure is described by Brønnum-Hansen.^18^

## Results

It is clear from table 1 that the prevalence of diabetes is higher among men than among women and higher for obese than for not obese and also that the educational gradient applies to both obese and not obese.

**Table 1:** Number of participants aged 30+ in health survey (DNHS) and prevalence of diabetes among not obese and obese by level of education (Denmark 2021)

		Men		Women	
		Number of participants in DNHS	Prevalence of diabetes	Number of participants in DNHS	Prevalence of diabetes
Body weight	Educational level	*N*	%	*N*	%
Not obese (BMI ≤ 30)	Short	8406	11.6	9743	8.3
	Medium	19 192	7.2	19 834	4.4
	Long	23 565	5.2	31 452	2.3
	All^a^	52 246	7.1	62 373	4.0
Obese (BMI > 30)	Short	3332	22.2	3313	18.1
	Medium	5844	16.4	5841	11.3
	Long	4408	14.4	6180	9.0
	All^a^	13 868	17.3	15 647	11.9

a Including people under education or with non-categorizable educations (12 059 individuals, 7.7%).

Tables 2 and 3 show the association between obesity and life expectancy and diabetes-free life expectancy at age 30 and 65, respectively. Life expectancy at age 30 for not obese men and women was 50.6 and 53.6 years, while it was 48.3 and 51.6 years for obese men and women (table 2). For people aged 65, the difference between not obese and obese was also clear, 1.2 and 1.0, respectively, for men and women (table 3). Regardless of whether they were obese or not, women could expect more years without and fewer years with diabetes than men.

**Table 2:** Life expectancy, expected lifetime without and with diabetes and proportion of diabetes-free life expectancy for not obese and obese individuals at age 30 (Denmark 2021)

		Life expectancy	Expected lifetime without diabetes	Expected lifetime with diabetes	Proportion of expected lifetime without diabetes
Body weight	Educational level	Years	Years (95% CI)	Years (95% CI)	% (95% CI)
Men					
Not obese (BMI ≤ 30)	Short	47.6	43.5 (43.2–43.8)	4.2 (3.9–4.4)	91.3 (90.7–91.9)
	Medium	50.7	47.6 (47.4–47.8)	3.1 (2.9–3.3)	93.9 (93.6–94.2)
	Long	53.3	50.4 (50.3–50.6)	2.8 (2.7–3.0)	94.7 (94.4–95.0)
	All^a^	50.6	47.3 (47.2–47.4)	3.2 (3.1–3.3)	93.6 (93.4–93.8)
Obese (BMI > 30)	Short	44.7	36.8 (36.2–37.4)	7.9 (7.3–8.5)	82.3 (81.0–83.7)
	Medium	48.5	41.4 (41.0–41.8)	7.1 (6.7–7.6)	85.3 (84.4–86.2)
	Long	51.7	44.5 (43.9–45.1)	7.1 (6.6–7.7)	86.2 (85.1–87.3)
	All^a^	48.3	40.9 (40.6–41.2)	7.4 (7.1–7.7)	84.7 (84.1–85.3)
Women					
Not obese (BMI ≤ 30)	Short	51.3	47.9 (47.7–48.2)	3.3 (3.1–3.6)	93.5 (92.9–94.0)
	Medium	54.0	51.7 (51.6–51.9)	2.2 (2.1–2.4)	95.9 95.6–96.2)
	Long	55.4	53.8 (53.7–54.0)	1.5 (1.4–1.6)	97.3 (97.0–97.5)
	All^a^	53.6	51.4 (51.3–51.5)	2.2 (2.1–2.3)	95.9 (95.8–96.1)
Obese (BMI > 30)	Short	48.6	41.3 (40.7–41.9)	7.3 (6.7–7.8)	85.1 (83.8–86.3)
	Medium	52.1	46.1 (45.6–46.6)	6.0 (5.5–6.5)	88.5 (87.6–89.5)
	Long	53.8	47.9 (47.3–48.5)	5.9 (5.3–6.6)	89.0 (87.7–90.1)
	All^a^	51.6	45.3 (45.0–45.6)	6.3 (6.0–6.6)	87.8 (87.2–88.3)

a Including people under education or with non-categorizable educations.

CI: confidence interval.

**Table 3:** Life expectancy, expected lifetime without and with diabetes and proportion of diabetes-free life expectancy for not obese and obese individuals at age 65 (Denmark 2021)

		Life expectancy	Expected lifetime without diabetes	Expected lifetime with diabetes	Proportion of expected lifetime without diabetes
Body weight	Educational level	Years	Years (95% CI)	Years (95% CI)	% (95% CI)
Men					
Not obese (BMI ≤ 30)	Short	16.9	14.3 (14.1–14.5)	2.6 (2.4–2.8)	84.6 (83.6–85.7)
	Medium	18.1	16.0 (15.9–16.1)	2.1 (2.0–2.2)	88.4 (87.6–89.1)
	Long	19.8	17.8 (17.6–17.9)	2.0 (1.9–2.2)	89.8 (89.1–90.5)
	All^a^	18.3	16.0 (15.9–16.1)	2.2 (2.2–2.3)	87.7 (87.3–88.2)
Obese (BMI > 30)	Short	15.6	10.8 (10.4–11.1)	4.8 (4.4–5.2)	69.2 (66.8–71.7)
	Medium	17.0	12.5 (12.2–12.9)	4.4 (4.1–4.8)	73.9 (71.8–76.0)
	Long	18.8	14.3 (13.8–14.8)	4.5 (4.0–5.0)	76.1 (73.4–78.9)
	All^a^	17.1	12.4 (12.2–12.7)	4.6 (4.4–4.9)	72.9 (71.5–74.2)
Women					
Not obese (BMI ≤ 30)	Short	19.4	17.3 (17.2–17.5)	2.1 (1.9–2.2)	89.4 (88.6–90.2)
	Medium	20.7	19.2 (19.0–19.3)	1.5 (1.4–1.6)	92.7 (92.1–93.3)
	Long	21.6	20.6 (20.4–20.7)	1.0 (0.9–1.2)	95.2 (94.6–95.8)
	All^a^	20.4	18.9 (18.8–19.0)	1.5 (1.5–1.6)	92.5 (92.1–92.9)
Obese (BMI > 30)	Short	18.2	14.0 (13.6–14.4)	4.2 (3.8–4.6)	76.9 (74.9–79.0)
	Medium	19.7	15.9 (15.4–16.3)	3.8 (3.4–4.3)	80.6 (78.3–82.9)
	Long	20.7	16.7 (16.1–17.3)	4.0 (3.4–4.6)	80.6 (77.6–83.5)
	All^a^	19.4	15.3 (15.1–15.5)	4.1 (3.8–4.3)	79.0 (77.8–80.3)

a Including people under education or with non-categorizable educations.

CI: confidence interval.

Life expectancy at age 30 for men with a short, medium and long education was 46.7, 50.2 and 53.0 years, respectively (data not shown). For women, life expectancy was 50.5, 53.6 and 55.1 years, respectively. Educational inequalities in life expectancy and diabetes-free life expectancies appear in table 2 (at age 30) and table 3 (at age 65). The difference in expected lifetime between not obese 30-year-old men and women with long and short education was 5.7 (53.3–47.6) years and 4.1 (55.4–51.3) years, respectively. For obese men and women, the difference was 7.0 (51.7–44.7) and 5.2 (53.8–48.6) years. Diabetes-free life expectancy differed by 6.9 (50.4–43.5) and 7.7 (44.5–36.8) years between not obese and obese 30-year-old men with a short and a long education (table 2). For women, the difference was 5.9 (53.8–47.9) and 6.6 (47.9–41.3) years, respectively. It appears from the not-overlapping CIs that all these differences were statistically significant. Table 3 shows similar patterns for the 65-years-old.

From table 2, it can be deduced that for 30-year-old men with a short education, the loss of life expectancy and diabetes-free life expectancy related to obesity were 2.9 and 6.7 years, respectively. The loss was almost the same for women with a short education (2.7 and 6.6 years). For people with a long education, the loss of life expectancy and years without diabetes was less: 1.6 and 5.9 years for both sexes. The loss of diabetes-free life expectancy related to obesity was lowest among women in the middle educational group, 5.6 years. These results point to an educational differential association between obesity and life expectancy and diabetes-free life expectancy. However, the loss of diabetes-free life expectancy within educational groups was almost equal for 65-year-olds, 3.5 years for men and 3.3 years for women with a short and a medium education and 3.9 years for women with a long education (table 3).

Tables 2 and 3 also show that despite reduced lifespan, expected lifetime with diabetes was longer among obese than among not obese individuals, which also meant, that the proportion of expected lifetime without diabetes was statistically significantly lower among obese than not obese people regardless of educational level. Furthermore, it appears from table 2 that despite the shorter lifespan, 30-year-old obese men with a short education can expect more years with diabetes (7.9 years) than obese men with a long education (7.1 years). The same association was seen for 30-year-old women. Table 3 shows a similar pattern for 65-year-olds.

## Discussion

An educational gradient in life expectancy and diabetes-free life expectancy was found in this study and, importantly, a marked difference between educational groups in diabetes-free life expectancy related to obesity. The impact of obesity seems most severe among people with a short education.

The prevalence of obesity is unevenly distributed across educational levels (table 1) and, like a large number of other conditions and health determinants, contributes to explaining differences in life expectancy and diabetes-free life expectancy between the educational groups. However, loss of life years and diabetes-free life expectancy within educational groups show socially unequal vulnerability to the health effects of obesity and a remarkably longer expected lifetime with diabetes among obese people with a short education. The design of the study did not allow for any conclusion on causality. However, Mathisen *et al*.^11^ found that differential exposure and susceptibility to being overweight/obese were both important mechanisms in the association between education and the incidence of type 2 diabetes. Bender *et al*.^19^ found that the joint effect of low education and diabetes increased the absolute and relative risk of receiving disability pension, but how obesity contributes to that mechanism has not been investigated, neither has the joint effects on mortality been investigated.

Because of the sex difference in the prevalence of obesity and diabetes, and mortality rates and the age-dependent differences between men and women in educational attainment, the estimates were stratified by sex. The proportion of expected lifetime without diabetes takes into account the female advantage in life expectancy, and for all levels of education the proportion was higher for women than for men, which is a counterexample of the well-known male–female health–mortality paradox.^20^

Quite a few studies have estimated socioeconomic differentials in health expectancy based on several health indicators.^21^ However, only a few health expectancy studies have combined the effects of socioeconomic position and risk factor exposure. The educational differential association between body weight and health of the Danish population has recently been examined by estimating health expectancies based on three indicators (disease-free life expectancy, disability-free life expectancy and lifetime in self-rated good health).^22^ The study focused on the loss of life expectancy and years of healthy life lost within educational groups and concluded that the impact of obesity did not differ significantly regardless of which of the three indicators of health expectancy were used. In the present study, however, a social gradient was seen for diabetes-free life expectancy at age 30 when the loss of healthy years within educational levels was compared.

While the number of health expectancy studies that stratify both by risk factor exposure and socioeconomic position is sparse, this is even more so for diabetes-free life expectancy studies that combine socioeconomic position and health determinants. To our knowledge, only the two studies mentioned in the introduction^7^^,^^8^ investigated the effect of obesity on diabetes-free life expectancy. Furthermore, only the simulation study by Bender *et al*.^8^ analysed social differentials and found that if the prevalence of overweight and obesity among people with short and medium education was reduced to that of people with a long education, life expectancy would increase by about three months among people with short and medium educations and reduce by almost 1-year lifetime with diabetes for women and 7 months for men.

Because educational-specific life tables were constructed from nationwide registers by linkage of information on the highest level of completed education and date of death at the individual level, the association between educational attainment and life expectancy is consistent and indisputable. The impact of obesity on life expectancy was calculated by use of sex- and age group-specific relative risk estimates which was based on a Danish study by Eriksen *et al*.^15^ Thus, the relative risks reflect population characteristics consistent with the data used in the present study. However, due to a lack of educational-, sex- and age-specific relative risk estimates, the impact of body weight on mortality was assumed not to differ between educational groups.

The self-reported information on education from DNHS might be somewhat overrated in comparison with registered information. Since health problems (inclusive diabetes) generally decrease with the level of education, this bias is expected to imply an increasing underestimation of healthy years by educational level. A similar effect of overrating body height and underrating body weight may be reflected in underreported obesity.

Bias introduced by differential response rates combined with differential health status might imply that diabetes-related health problems were more underreported among people with a short education than people with longer educations. This bias is assumed to be reduced by using weights that account for different sampling probabilities and differential non-response.^13^

Longitudinal data are preferred for most epidemiological studies as for analyses of health expectancy to capture the dynamics of demographic developments and changes in public health. But sufficient richness and diversity of such data are rare, and despite the lack of data to allow the analysis of these dynamics, the more static and less data-intensive Sullivan method is useful if (period) life tables by combined determinants can be constructed (e.g. combinations of educational level and risk factor exposure).

## Conclusion and implications

The study demonstrates the need for more efforts to prevent overweight and obesity with special attention on people with short education. However, interventions targeting high-risk individuals have only a minor impact on the obesity epidemic and should be implemented with structural interventions. Successful interventions face major challenges because strong restrictions are needed to protect the population from the harmful products of the food industry. Structural prevention initiatives that can contribute to reducing social inequalities in health include significant increases in taxes on unhealthy products, reducing access and accessibility, stopping the marketing and sponsorship of harmful products and creating attractive facilities in the local environment for physical activity.^23^^,^^24^

## Supplementary Material

ckad153_Supplementary_Data

## Data Availability

Microdata from the national registers accessed from Statistics Denmark and data from the Danish National Health Survey are not publicly available. The Supplementary material includes aggregated data for the construction of life tables.

## References

[1] Tomic D , Morton JI, Chen L, et al Lifetime risk, life expectancy, and years of life lost to type 2 diabetes in 23 high-income jurisdictions: a multinational, population-based study. Lancet Diabetes Endocrinol 2022;10:795–803. 36183736 10.1016/S2213-8587(22)00252-2 PMC10988609

[2] Nwaneri C , Cooper H, Bowen-Jones D. Mortality in type 2 diabetes mellitus: magnitude of the evidence from a systematic review and meta-analysis. Diab Vasc Dis 2013;13:192–207.

[3] Espelt A , Borrell C, Roskam AJ, et al Socioeconomic inequalities in diabetes mellitus across Europe at the beginning of the 21st century. Diabetologia 2008;51:1971–9. 18779946 10.1007/s00125-008-1146-1

[4] Abdullah A , Peeters A, de Courten M, et al The magnitude of association between overweight and obesity and the risk of diabetes: a meta-analysis of prospective cohort studies. Diabetes Res Clin Pract 2010;89:309–19. 20493574 10.1016/j.diabres.2010.04.012

[5] Magliano DJ , Shaw JE, Shortreed SM, et al Lifetime risk and projected population prevalence of diabetes. Diabetologia 2008;51:2179–86. 18810385 10.1007/s00125-008-1150-5

[6] Andrade F. Estimating diabetes and diabetes-free life expectancy in Mexico and seven major cities in Latin America and the Caribbean. Rev Panam Salud Publica 2009;26:9–16. 19814876 10.1590/s1020-49892009000700002 PMC4059400

[7] Cunningham SA , Riosmena F, Wang J, et al Decreases in diabetes-free life expectancy in the U.S. and the role of obesity. Diabetes Care 2011;34:2225–30. 21949220 10.2337/dc11-0462 PMC3177736

[8] Bender AM , Sørensen J, Diderichsen F, et al A health inequality impact assessment from reduction in overweight and obesity. BMC Public Health 2020;20:1823. 33256647 10.1186/s12889-020-09831-x PMC7706236

[9] Sikdar KC , Wang PP, MacDonald D, et al Diabetes and its impact on health-related quality of life: a life table analysis. Qual Life Res 2010;19:781–7. 20349211 10.1007/s11136-010-9641-5

[10] Tönnies T , Baumert J, Heidemann C, et al Diabetes free life expectancy and years of life lost associated with type 2 diabetes: projected trends in Germany between 2015 and 2040. Popul Health Metr 2021;19:38. 34635124 10.1186/s12963-021-00266-z PMC8507142

[11] Mathisen J , Jensen AKG, Andersen I, et al Education and incident type 2 diabetes: quantifying the impact of differential exposure and susceptibility to being overweight or obese. Diabetologia 2020;63:1764–74. 32361776 10.1007/s00125-020-05150-3

[12] Jensen VM , Rasmussen AW. Danish education registers. Scand J Public Health 2011;39:91–4. 21775362 10.1177/1403494810394715

[13] Rosendahl H , Davidsen M, Møller SR, et al *Danes' Health: The National Health Profile 2021* [Danskernes sundhed: Den Nationale Sundhedsprofil 2021]*.* Danish Health Authority, Copenhagen (in Danish), 2022. https://www.sst.dk/-/media/Udgivelser/2022/Sundhedsprofil/Sundhedsprofilen.ashx?sc_lang=da&hash=5C9A9A81483F6C987D5651976B72ECB2 (17 August 2023, date last accessed).

[14] Carstensen B , Rønn PF, Jørgensen ME. Prevalence, incidence and mortality of type 1 and type 2 diabetes in Denmark 1996-2016. BMJ Open Diab Res Care 2020;8:e001071. 10.1136/bmjdrc-2019-001071 PMC7265004 32475839

[15] Eriksen L , Davidsen M, Jensen HAR, et al *The Burden of Disease in Denmark – Risk Factors*. [Sygdomsbyrden i Danmark—risikofaktorer]. Statens Institut for Folkesundhed, Syddansk Universitet for Sundhedsstyrelsen (in Danish). 2016. https://www.sst.dk/-/media/Udgivelser/2016/Sygdomsbyrden-i-Danmark_2016.ashx?la=da&hash=6C5DF5B672D84689EFC4EB7BAB1C94C687FE7C61 (17 August 2023, date last accessed).

[16] Sullivan DF. A single index of mortality and morbidity. Health Serv. Ment. Health Adm. Health Rep 1971;86:347–54. PMC1937122 5554262

[17] Jagger C , Van Oyen H, Robine JM. *Health Expectancy Calculation by the Sullivan Method: A Practical Guide*. Technical report, 4th edn. EHLEIS, 2014. https://reves.site.ined.fr/fichier/s_rubrique/20182/sullivan.guide.pre.final.oct2014.en.pdf (17 August 2023, date last accessed).

[18] Brønnum-Hansen H. Assessing the impact of risk factors on health expectancy. In: Jagger C, Crimmins EM, Saito Y, et al, editors. International Handbook of Health Expectancies. Cham, Switzerland: Springer, 2020: 123–7.

[19] Bender AM , Vrangbæk K, Lange T, et al Joint effects of educational attainment, type 2 diabetes and coexisting morbidity on disability pension: results from a longitudinal, nationwide, register-based study. Diabetologia 2021;64:2762–72. 34518897 10.1007/s00125-021-05559-4

[20] Di Lego V , Lazarevič P, Luy M. The male-female health-mortality paradox. In: Gu D, Dupre ME, editors. Encyclopedia of Gerontology and Population Aging. Cham, Switzerland: Springer, 2020.

[21] Cambois E , Brønnum-Hansen H, Hayward M, et alMonitoring social differentials in health expectancies. In: Jagger C, Crimmins EM, Saito Y, et al editors. International Handbook of Health Expectancies. Cham, Switzerland: Springer, 2020: 45–66.

[22] Brønnum-Hansen H , Davidsen M. Social differentials in the impact of risk factor exposures on life expectancy and health expectancy. J Public Health (Berl) 2022;31:1387–1399. 10.1007/s10389-022-01724-0.

[23] Marteau TM , White M, Rutter H, et al Increasing healthy life expectancy equitably in England by 5 years by 2035: could it be achieved? Lancet 2019;393:2571–3. 31258113 10.1016/S0140-6736(19)31510-7

[24] Danish Health Authority. *Efforts Against Inequality in Health*. [Indsatser mod ulighed i sundhed]. Copenhagen (in Danish): Danish Health Authority, 2020.

