# Supplementary material for: Impact of the association between education and obesity on diabetes-free life expectancy
Source: Eur J Public Health. 2023 Aug 23;33(6):968–73. doi: 10.1093/eurpub/ckad153 (PMC10710352; doi:10.1093/eurpub/ckad153)
Supplement: ckad153_Supplementary_Data [file ckad153_supplementary_data.zip › ckad153_Supplementary_Data/ejph-2023-02-om-0098-File006.pdf]

Basic aggregated data for life table construction by education and BMI

	Death rates, Men			Death rates, Women		
	Educational level			Educational level		
Age	Short	Medium	Long	Short	Medium	Long
30-34	0.00509	0.00213	0.00096	0.00289	0.00112	0.00086
35-39	0.01007	0.00375	0.00187	0.00673	0.00173	0.00139
40-44	0.01361	0.00518	0.00267	0.00963	0.00391	0.00207
45-49	0.02110	0.00912	0.00451	0.01451	0.00551	0.00381
50-54	0.02998	0.01398	0.00816	0.01909	0.01011	0.00597
55-59	0.04853	0.02514	0.01487	0.03302	0.01662	0.01271
60-64	0.06867	0.04646	0.02572	0.04331	0.03083	0.01917
65-69	0.11502	0.07812	0.04690	0.07088	0.04850	0.03318
70-74	0.15332	0.11343	0.08235	0.09744	0.07058	0.05502
75-79	0.21402	0.19292	0.15076	0.15589	0.11873	0.10405
80-84	0.32369	0.31314	0.25154	0.25153	0.21171	0.18319
85-89	0.54035	0.51450	0.46978	0.40995	0.39641	0.36180
90+	0.75941	0.76442	0.71969	0.62895	0.61526	0.57205

Age groups fitting RR estimates	Prevalence of obesity* (%), Men			Prevalence of obesity* (%), Women		
	Educational level			Educational level		
	Short	Medium	Long	Short	Medium	Long
16-34	28.3	25.8	12.1	32.4	30.8	15.0
35-64	31.8	24.7	15.8	30.1	25.2	17.5
65-74	28.8	22.8	17.6	26.7	19.4	14.8
75+	20.5	16.5	12.2	19.0	13.4	10.9

* Obesity: BMI > 30

Estimates of relative risks for death from obesity by age and sex

	Mortality relative risks (obese relative to not obese)	
Age groups	Men	Women
16-34	1.25	1.30
35-64	1.50	1.59
65-74	1.29	1.29
75+	1.16	1.21

Source: Eriksen et al.[15] (Table 9.4.1)

More details about the method for calculating health expectancy by risk factor exposure is described in [18]: Brønnum-Hansen H. Assessing the Impact of Risk Factors on Health Expectancy. In: Jagger C, Crimmins EM, Saito Y, et al., eds. International Handbook of Health Expectancies. Cham, Switzerland: Springer, 2020: 123–7
